# Hemoglobin estimation by the HemoCue^® ^portable hemoglobin photometer in a resource poor setting

**DOI:** 10.1186/1472-6890-11-5

**Published:** 2011-04-21

**Authors:** Bernard Nkrumah, Samuel Blay Nguah, Nimako Sarpong, Denise Dekker, Ali Idriss, Juergen May, Yaw Adu-Sarkodie

**Affiliations:** 1 Kumasi Centre for Collaborative Research in tropical Medicine, Kumasi, Ghana; 2 Komfo Anokye Teaching Hospital, Kumasi, Ghana; 3 Malaria Research Centre, Agogo, Ghana; 4 Bernhard Nocht Institute for Tropical Medicine and Hygiene, Hamburg, Germany; 5 Kwame Nkrumah University of Science and Technology, Kumasi, Ghana

## Abstract

**Background:**

In resource poor settings where automated hematology analyzers are not available, the Cyanmethemoglobin method is often used. This method though cheaper, takes more time. In blood donations, the semi-quantitative gravimetric copper sulfate method which is very easy and inexpensive may be used but does not provide an acceptable degree of accuracy. The HemoCue^® ^hemoglobin photometer has been used for these purposes. This study was conducted to generate data to support or refute its use as a point-of-care device for hemoglobin estimation in mobile blood donations and critical care areas in health facilities.

**Method:**

EDTA blood was collected from study participants drawn from five groups: pre-school children, school children, pregnant women, non-pregnant women and men. Blood collected was immediately processed to estimate the hemoglobin concentration using three different methods (HemoCue^®^, Sysmex KX21N and Cyanmethemoglobin). Agreement between the test methods was assessed by the method of Bland and Altman. The Intraclass correlation coefficient (ICC) was used to determine the within subject variability of measured hemoglobin.

**Results:**

Of 398 subjects, 42% were males with the overall mean age being 19.4 years. The overall mean hemoglobin as estimated by each method was 10.4 g/dl for HemoCue, 10.3 g/dl for Sysmex KX21N and 10.3 g/dl for Cyanmethemoglobin. Pairwise analysis revealed that the hemoglobin determined by the HemoCue method was higher than that measured by the KX21N and Cyanmethemoglobin. Comparing the hemoglobin determined by the HemoCue to Cyanmethemoglobin, the concordance correlation coefficient was 0.995 (95% CI: 0.994-0.996, p < 0.001). The Bland and Altman's limit of agreement was -0.389 - 0.644 g/dl with the mean difference being 0.127 (95% CI: 0.102-0.153) and a non-significant difference in variability between the two measurements (p = 0.843). After adjusting to assess the effect of other possible confounders such as sex, age and category of person, there was no significant difference in the hemoglobin determined by the HemoCue compared to Cyanmethemoglobin (coef = -0.127, 95% CI: -0.379 - 0.634).

**Conclusion:**

Hemoglobin determined by the HemoCue method is comparable to that determined by the other methods. The HemoCue photometer is therefore recommended for use as on-the-spot device for determining hemoglobin in resource poor setting.

## Background

Hemoglobin (Hb) assessments are the most reliable indicator widely used to screen individuals for anaemia, to draw inferences about the iron status of populations and to evaluate responses to nutritional interventions [1]. The blood Hb concentration is an important variable directing transfusion therapy in patients suffering major blood loss due to accidents, surgery, labour and many other critical conditions [2]. Hb concentration is measured routinely using automated hematology analyzers, such as those produced by the Sysmex Corporation (Kobe, Japan). Although these counters are very accurate and reliable, they are expensive and transport of the samples to the laboratory delays the process which may delay treatment, resulting in preventable deaths [3]. In resource poor settings where automated hematology analyzers are not available, the Cyanmethemoglobin method is often used. Hb estimation by this method though cheaper than the automated method takes more time [4]. In blood donations, the semi-quantitative gravimetric copper sulfate method which is very easy and inexpensive may be used, but does not provide an acceptable degree of accuracy [5,6]. The HemoCue^® ^Hb photometer has been widely used for these purposes in recent years [7] because it is portable, requires only a small sample of capillary/venous blood, is relatively inexpensive and simple to use, does not require access to refrigeration or even electricity, and gives immediate, digitally displayed results [3]. However, data to support the use of this device for all categories of patients in resource limited areas of Ghana is not available. This study was therefore conducted to generate data to support or refute its use as a point-of-care device for Hb estimation, mobile blood donations and critical care areas in health facilities.

## Methods

### Study Subjects

A total of 398 consented study patients were involved in this study. They were placed in five groups: pre-school children (subjects within 1-4 years), school children (subjects within 5-14 years), pregnant women (No age limit), non-pregnant women (female subjects within 15-59 years) and men (male subjects within 15-59 years).

### Study Design

This was a hospital-based study conducted at the Agogo Presbyterian Hospital in the Asante Akim North District (AAND), Ashanti Region, Ghana. Blood samples were collected from consented participants within the study group reporting to the hospital after study procedures had been explained to them. EDTA blood samples taken from patients for routine laboratory investigations were used for the study thus no extra blood was collected except for patients in which EDTA blood was not taken, in which case venous blood was taken from the arm. Venous blood collected from participants was processed to estimate their Hb content using three different methods (HemoCue^®^, Sysmex KX21N and Cyanmethemoglobin).

### Study Site

The Agogo Presbyterian Hospital in Agogo was the study site. This is the major hospital serving the Asante-Akim North District and other parts of the Ashanti region. The main departments of the hospital are the Children's, Casualty, Surgical, Medical, Obstetrics and Gynecology, Eye and the laboratory Departments. The laboratory department of the hospital offers diagnostic as well as research services. The department is fully equipped and has functional Microbiology, Hematology, Parasitology and Clinical Chemistry departments. The laboratory participates in various External Quality Assessment programmes one of which is the United Kingdom National External Quality Assessment Service (UK NEQAS) in hematology. Among the most prevalent diseases in the district are malaria, buruli ulcer, typhoid fever and tuberculosis.

### Ethical Approval

Ethical approval for this study was obtained from the Committee on Human Research, Publication and Ethics (CHRPE), of the School of Medical Sciences, KNUST-Kumasi. After information and appropriate explanations, adults or parents/legal guardians of children willing to participate in the study had to give their consent by appending their signature or thumbprint to the informed consent form before any study related procedures were done.

### HemoCue^® ^Portable Photometer

The HemoCue^® ^B-Hemoglobin system (HemoCue AB, Ängelholm, Sweden) consists of disposable microcuvettes containing reagent in a dry form and a single purpose designed photometer. The microcuvettes were stored in a dry place at room temperature. Once opened, they were tightly closed and stored at the same conditions to maintain their integrity and shelf life. The reaction in the microcuvette is a modified azide-methemoglobin reaction. Sodium deoxycholate haemolyses erythrocytes and hemoglobin is released. Sodium nitrite converts hemoglobin to methemoglobin which, together with sodium azide, gives azidemethemoglobin. The absorbance is measured at two wavelengths (570 nm and 880 nm) in order to compensate for turbidity in the sample. The test was performed as stated by the manufacturer [8].

### Sysmex KX21N Hematology Analyzer

The Sysmex KX21N (Sysmex Corporation, Kobe, Japan) is an automated blood cell counter intended for in vitro diagnostic use in clinical laboratories. It is a compact, fully automated hematology analyzer with simultaneous analysis of 18 parameters in whole blood mode and capillary blood mode. It measures the hemoglobin concentration using a non-cyanide hemoglobin method (STROMATOLYSER WH). The instrument has been proven to provide accurate and reliable results including hemoglobin concentrations [9,10]. The test was performed as stated in the manufacturer's manual [11].

### Cyanmethemoglobin Method

The principle of this method lies in conversion of hemoglobin to cyanmethemoglobin by the addition of Potassium cyanide and ferricyanide whose absorbance is measured at 540 nm in a photoelectric calorimeter against a standard solution. The test was performed as outlined by Bhaskaram et al [12].

### Quality Control

The function of the HemoCue photometer was checked on a daily basis by measuring the control cuvette (Serial no: 0214-003 071) and a standard of known concentration. A three set EIGHTCHECK-3WP controls (Low, Normal and High) were run daily to ensure the function of the Sysmex KX21N. For the cyanmethemoglobin, a hemoglobin standard of known concentration was tested daily. Samples were only processed when the QC material had passed.

### Statistical Analysis

Data was double-entered into a predesigned electronic database using Epi info version 6.04 (Center for Disease Control, Atlanta, GA, USA) and cleaned on a regular basis. It was then exported to *Stata/*SE10.1 statistical software (Stata Corporation, Texas USA) for analysis. A pairwise t-test was used to compare the HemoCue and both the Sysmex and cyanmethemoglobin. Agreement between the test methods was assessed by the method of Bland and Altman [13], where the mean, standard deviation and limit of agreement of paired results were calculated. Figures plotting difference against the average were graphed as Bland and Altman recommended [13]. The Intraclass correlation coefficient (ICC) was used to determine the within subject variability of measured hemoglobin whiles the concordance correlation coefficient (CCC) was used to measure the agreement between the three methods.

## Results

The study was carried out from August to December 2010. Three hundred and ninety eight consented study subjects were recruited for the study. Of these, school children constituted the highest population (22.4%) whilst adult males constituted the least (16.6%). Overall, there were fewer males (42%) than females. Whilst adult males had the highest mean age of 34.3 years (range: 16-59 years), pre-school children recorded the least with 2.3 years (range: 1-4 years), with the overall mean age being 19.4 years (range: 1-64 years) (Table 1).

**Table 1:** Summary of subject categories in the various subgroups

	Study Subgroup					
	
	School Children	Pre-School Children	Pregnant Women	Adult Men	Non-pregnant Women	Overall
Number (%)	89 (22.4)	87 (21.9)	73 (18.3)	66 (16.6)	83 (20.8)	100
Male n (%)	51 (57.5)	50 (57.5)	0	66 (100.0)	0	167 (42.0)
**Age in years**						
Mean (SD)	7.8 (2.3)	2.3 (1.1)	27.2 (5.8)	34.3 (12.3)	30.9 (11.7)	19.4 (15.2)
95% CI	7.3-8.3	2.1-2.6	25.8-28.6	31.2-37.3	28.4-33.5	17.9-20.9
Range	5-14	1-4	17-41	16-59	12-64	1-64
**Hb(g/dl) by HemoCue**						
Mean (SD)	9.8 (3.1)	9.3 (2.9)	11.1 (1.7)	11.4 (3.3)	10.8 (2.5)	10.4 (2.8)
95% CI	9.1-10.4	8.7-9.9	10.7-11.5	10.6-12.2	10.3-11.3	10.1-10.7
Range	3.3-20.3	2.7-16.8	5.6-16.6	3.2-16.8	2.9-16.7	2.7-20.3
**Hb(g/dl) by KX21N**						
Mean (SD)	9.6 (3.1)	9.1 (2.9)	10.9 (1.6)	11.3 (3.4)	10.7 (2.4)	10.2 (2.9)
95% CI	8.9-10.3	8.5-9.8	10.5-11.3	10.4-12.1	10.1-11.2	10.0-10.5
Range	2.7-20.4	2.4-16.9	5.5-16.0	3.3-16.9	2.9-16.6	2.4-20.4
**Hb(g/dl) by Cyanmethemoglobin**						
Mean (SD)	9.6 (3.1)	9.2 (2.9)	11.0 (1.7)	11.3 (3.3)	10.7 (2.5)	10.3 (2.8)
95% CI	9.0-10.3	8.6-9.8	10.6-11.4	10.4-12.1	10.1-11.2	10.0-10.5
Range	2.9-20.4	2.5-16.4	5.5-16.3	3.0-16.7	2.7-16.5	2.5-20.4

There was a significant but marginal increase in hemoglobin with age (0.03 g/dl/year, p < 0.001). Pre-school and school children recorded the lowest mean hemoglobin concentration of 9.2 g/dl (SD: 2.9) and 9.7 g/dl (SD: 3.1) respectively. This was followed by non-pregnant and pregnant women with mean hemoglobin of 10.7 g/dl (SD: 2.4) and 11.0 g/dl (SD: 1.7) respectively. Adult males had the highest mean hemoglobin of 11.3 g/dl (SD: 3.3). The mean hemoglobin concentration of males was 0.5 g/dl (95%CI: 0.1 to 0.8 g/dl, p = 0.01), higher than that of the females. This effect persisted even after correcting for the age, method used to estimate hemoglobin and study sub-population. Pairwise analysis revealed that the hemoglobin determined by the HemoCue method was higher than that measured by the KX21N and Cyanmethemoglobin with mean differences of 0.15 g/dl (95% CI: 0.12 to 0.17, p < 0.001) and 0.13 g/dl (95%CI: 0.10 to 0.15, p < 0.001) respectively. That measured by the KX21N and Cyanmethemoglobin methods were however not significantly different (0.02 g/dl, 95%CI: 0.00 to 0.04, p = 0.10).

**Figure 1:**
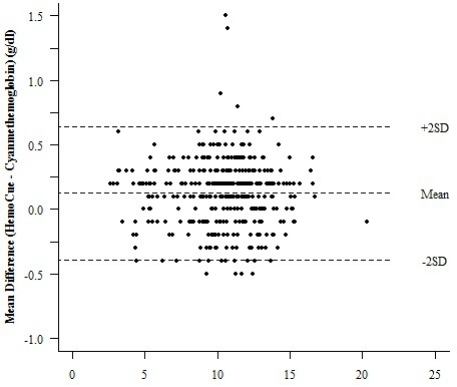
**Bland and Altman plot for HemoCue and Cyanmethemoglobin**. Mean of HemoCue and Cyanmethemoglobin (g/dl).

**Figure 2:**
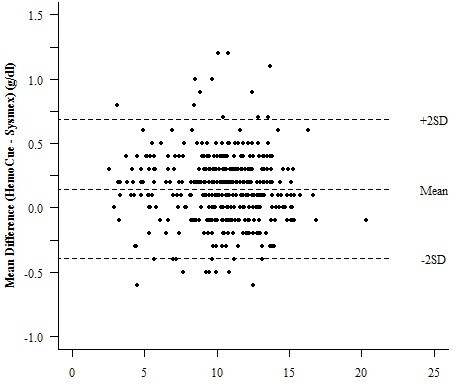
**Bland and Altman plot for HemoCue and Sysmex KX21N**. Mean of HemoCue and Sysmex (g/dl).

There was near perfect concordance correlation between the hemoglobin concentrations determined by the HemoCue and Cyanmethemoglobin methods (rho = 0.995) as well as HemoCue and KX21N (0.994). These were not significantly different for that between KX21N and the Cyanmethemoglobin method (0.997) (Table 2). Comparing the hemoglobin concentration determined by the HemoCue and Cyanmethemoglobin, the Bland and Altman's limit of agreement was from -0.39 to 0.64 g/dl with the mean difference being 0.13, and a non-significant difference in variability between the two measurements (p = 0.843). For HemoCue and KX21N, the Bland and Altman's limit of agreement was from -0.39 to 0.69 g/dl with a mean difference of 0.15 and a non-significant difference in variability between the two measurements (p = 0.391) (Figure 1, 2).

**Table 2:** Bland and Altman methods comparison

	Hemo^¥ ^vrs Cyan*	**Hemo vrs KX21N**	**Cyan vrs KX21N**
	
Bland and Altman			
Limit of agreement (p-value)	- 0.39 to 0.64 (0.843)	- 0.39 to 0.69 (0.39)	- 0.45 to 0.49 (0.441)
Mean difference (95%CI)	0.13 (0.10 to 0.15)	0.15 (0.120 to 0.174)	0.02 (-0.00 to 0.04)
Concordance Correlation Coefficient			
rho (95% CI)	0.995 (0.994 to 0.996)	0.994 (0.993 to 0.995)	0.997 (0.996 to 0.997)
p-value	<0.001	<0.001	<0.001

¥ HemoCue * Cyanmethemoglobin

The within subject variability of measured hemoglobin was very minimal with high intraclass correlation coefficient for all the various subgroups (Table 3). The lowest intraclass coefficient was in pregnant women (0.986) and the highest was in school children (0.997). These coefficients were also associated with high estimated reliabilities ranging between 0.995 and 0.999.

**Table 3:** Within subject analysis of measured Hb for various study populations

		Study Subgroup				
	
		School Children	Pre-School Children	Pregnant Women	Adult Men	Non-pregnant Women
Intraclass correlation						
	Coefficient	0.997	0.995	0.986	0.996	0.993
	95% CI	0.996 to 0.998	0.994 to 0.997	0.980 to 0.991	0.995 to 0.998	0.990 to 0.995
Estimated Reliability		0.999	0.998	0.995	0.999	0.998

After adjusting to assess the effect of other possible confounders such as sex, age and category of person, there was no significant difference in the hemoglobin determined by the HemoCue compared to Cyanmethemoglobin (coef = -0.127, 95%CI: -0.379 - 0.634) and that by HemoCue compared to Sysmex KX21N (coef = -0.147, 95%CI: -0.676 - 0.382).

## Discussion

Accurate determination of hemoglobin concentration is a common element in assessing the extent of anemia and making a decision whether transfusion is necessary or not. This decision should be made based on reliable and rapidly assessed laboratory tests. In settings where a central laboratory is used for the purposes of testing and transfusion monitoring, the time loss for blood sample transportation create delays which may lead to the loss of lives[3,12].

The HemoCue is a portable device for measuring hemoglobin concentration [14] and it requires very little staff training thus making it a very useful tool in resource limited areas, critical care areas such as the theatre, pediatric ward, maternity, intensive care units etc as well as at field conditions since it can easily be transported. In this study, we compared it with the Cyanmethemoglobin method and the Sysmex KX21N used in the laboratory. We found no significant differences in the hemoglobin concentrations determined by all the three methods, with no perceptible trend for disagreement with high or low values. The three devices had comparable concordance correlation coefficient, limits of agreement and intraclass correlation coefficients thus supporting the use of the HemoCue portable photometer among the various study groups in our study setting.

Our study is in agreement with other studies conducted in other settings to support the use of the device. These include the studies conducted by von Schenck et al [15], Van de Louw et al; among patients with gastrointestinal bleeding [16], Rippmann et al; among surgical patients [17], Bridges et al; on repeated measurement of one sample [7], Neville; within urban general practice [18], Rechner et al; among neonates [19], Lardi et al; among patients undergoing aortic surgery in the theatre [14], Sari et al; among Indonesian mothers [4] and Radtke et al; among blood donors [20].

Nevertheless, other studies such as that conducted by Zhou et al; among pregnant women in a higher altitude area of Tibet, China [21] and Bhaskaram et al; among apparently healthy children of 1-6 years [12] do not support the use of the HemoCue in their various study populations.

The cyanrnethemoglobin method is a widely used method of estimating Hb. However, several reports indicate that results obtained using this method could be imprecise due to a number of factors: turbidity of the blood and the large dilution of the sample (20 μL of blood in 5 mL of Drabkin's solution) [22-24], it requires skillful technical operations in terms of accurate uptake of the blood volume into a calibrated hemoglobin pipette, careful mixing of the sample with the Drabkin's solution, measurement of absorbance in the photometer and calculation of actual value from a systematically constructed standard graph [12]. All these manual operations are time consuming and make the method unsuitable for large scale field/community studies. It is also well documented that conditions such as hyperlipemia [23] and Waldenstrom's macroglobulinemia [25] can cause falsely high results for hemoglobin in the filter photometer method such as the cyanmethemoglobin method.

The HemoCue is a portable machine which directly measures Hb from an undiluted blood sample [8] and background turbidity of the samples are corrected due to the measurement of two-wave lengths [15]. This method is quicker (60 seconds), simple to operate thus making it faster with a shorter turn-around time. In addition, it requires less blood (10 μL), is cost effective and a more accurate method [12,16,19] as well as having other properties as stated by Sari et al [4] and Sawant et al [26]. Issues of cost have also been being reported as a factor to take into account when considering Hb estimation with the HemoCue [4,18,26]. The mechanical filling of the cuvettes with 10 μl of the blood by capillary action avoids several manual errors which often occur in the collection, dilution and measurement of the sample in the cyanmethemoglobin method. Nevertheless, the presence of air bubbles, excess blood on the back of the cuvettes, over filling of the cuvettes and insufficient mixing of the samples may lead to erroneous results. These factors even though reduced to the barest minimum might have contributed to the higher Hb concentration observed in our study as also reported in other studies [14,15,17,18]. However, by offering adequate training on the technique of proper filling the cuvettes to avoid air bubbles and over filling, mixing samples adequately but gently and wiping off excess blood from the cuvettes without reducing the blood quantity, these errors can be easily avoided.

## Conclusion

Hemoglobin determined by the HemoCue method is comparable to that determined by both the Cyanmethemoglobin and Sysmex KX21N methods. The HemoCue photometer is therefore recommended for use as on-the-spot device for determining hemoglobin in resource limited areas as well as critical care areas of health facilities.

## Competing interests

The authors declare that they have no competing interests.

## Authors' contributions

BN planned, carried out the study and headed the drafting of the manuscript. SBN carried out the statistical interpretation and co-headed the drafting the manuscript. NS organized the day-to-day work as study coordinator. DD organized and contributed to the study. AI carried out the study and contributed in drafting of the manuscript. JM and YAS supervised the study and contributed to drafting the manuscript. All authors have read and approved the final manuscript. Financial support for this study was given by a Swiss Foundation.

## Pre-publication history

The pre-publication history for this paper can be accessed here:

http://www.biomedcentral.com/1472-6890/11/5/prepub
